# The two-domain architecture of LAMP2A within the lysosomal lumen regulates its interaction with HSPA8/Hsc70

**DOI:** 10.1080/27694127.2022.2069968

**Published:** 2022-05-01

**Authors:** Yuta Ikami, Kazue Terasawa, Tetsuro Watabe, Shigeyuki Yokoyama, Miki Hara-Yokoyama

**Affiliations:** a Department of Oral and Maxillofacial Surgery, Graduate School of Medical and Dental Sciences, Tokyo Medical and Dental University (TMDU), Yushima 1-5-45, Bunkyo-ku, Tokyo 113-8549, Japan; b Department of Biochemistry, Graduate School of Medical and Dental Sciences, Tokyo Medical and Dental University (TMDU), Yushima 1-5-45, Bunkyo-ku, Tokyo 113-8549, Japan; c RIKEN Cluster for Science, Technology and Innovation Hub, 1-7-22 Suehiro-cho, Tsurumi-ku, Yokohama 230-0045, Japan

**Keywords:** Chaperone-mediated autophagy, expanded genetic codes, HSPA8, LAMP2A, site-specific photo-crosslinking, site-specific steric hindrance

## Abstract

Chaperone-mediated autophagy (CMA) is a unique proteolytic pathway, in which cytoplasmic proteins recognized by HSPA8/Hsc70 (heat shock protein 8) are transported into lysosomes for degradation. LAMP2A (lysosomal-associated membrane protein 2A) recruits the substrate/chaperone complex to the lysosome membrane. The structure of LAMP2A comprises a large lumenal domain composed of two homologous subdomains (N-domain and C-domain, both with the β-prism fold), a transmembrane domain, and a short cytoplasmic tail. Although the homophilic interaction between LAMP2A molecules and the interaction between HSPA8 and LAMP2A have been suggested, whether the interactions are direct or mediated by other molecules has remained to be elucidated. We investigated the interactions by using expanded genetic code technologies that generate photo-crosslinking and/or steric hindrance at specified interfaces in mammalian cells. The results suggested that LAMP2A molecules assemble by facing each other with one side of the β-prism in their C-domains. We also detected the photo-crosslinking between the cytoplasmic tail of LAMP2A and HSPA8, revealing this direct interaction. We found that the truncation of the N-domain reduced the amount of HSPA8 that coimmunoprecipitates with LAMP2A. Our present results suggest that the two-domain architecture of the lumenal domains of LAMP2A underlies the interaction with HSPA8 at the cytoplasmic surface of the lysosome.

Chaperone-mediated autophagy (CMA) is a unique pathway, in which cytosolic proteins recognized by HSPA8/Hsc70 (heat shock protein 8) are incorporated into the lysosome for degradation (Fig. 1A). CMA regulates a variety of intracellular functions by clearing damaged proteins and degrading functional proteins. Decreases and excessive increases of CMA underlie diseases such as neurodegeneration and cancer, respectively, in aging and pathological settings.

**Figure 1.**
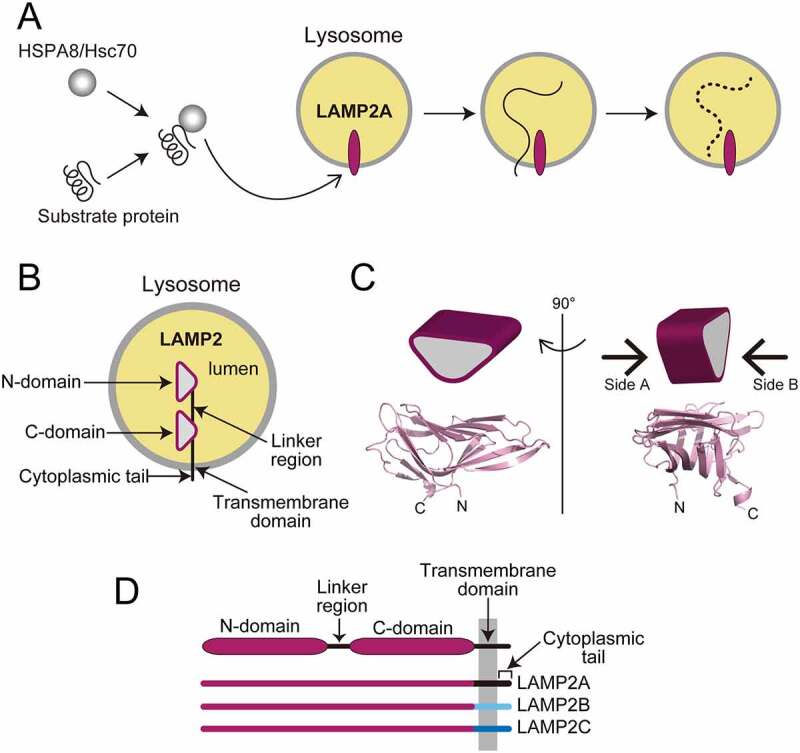
The role of LAMP2A in chaperone-mediated autophagy (**A**) and the domain structure of LAMP2A (**B-D**). (**B**) An orientation of the large lumenal domain of a LAMP2A molecule, composed of two homologous subdomains. (**C**) A schematic β-prism shape and the structure of the C-domain of LAMP1 (5GV0) are shown. N: N terminus of the C-domain; C: C terminus of the C-domain. (**D**) The C-terminal regions are distinct among LAMP2A, LAMP2B and LAMP2C due to alternative splicing.

In the initial step of CMA, LAMP2A (lysosomal-associated membrane protein 2A) acts as a scaffold for the substrate-chaperone complex on the lysosomal surface (Fig. 1A). LAMP2 and LAMP1 are the most abundant protein components of lysosome membranes. The lumenal domain of LAMP2 is composed of two parts, the membrane-distal domain (the N-domain) and the membrane-proximal domain (the C-domain). Both domains have the β-prism fold, a triangular prism composed of β-sheets, and are connected by the linker region (Fig. 1B, C). LAMP2A is one of the three LAMP2 isotypes—LAMP2A, LAMP2B, and LAMP2C—which have common lumenal domains and differ primarily in the sequences of their transmembrane and cytoplasmic tails (Fig. 1D). The specific involvement of LAMP2A in CMA suggests the importance of the cytoplasmic tail for CMA. Nevertheless, the molecular basis by which LAMP2A recruits the substrate-chaperone complex to the lysosome has not been sufficiently elucidated.

Several biochemical approaches have supported the homophilic interaction of LAMP2A. However, when the N- and C-domains of LAMP2A are prepared as secreted proteins, both domains exist as monomers. Therefore, these lumenal domains in isolation are unable to associate with themselves, suggesting that the transmembrane structures are required for the multimerization of LAMP2A.

To investigate the molecular interactions of the CMA components while maintaining their localization, we used site-specific photo-crosslinking and steric hindrance in mammalian cells (Fig. 2A). With this method, a non-natural amino acid (*p*-benzoyl-l-phenylalanine [*p*Bpa] or *N*^ε^-allyloxycarbonyl-l-lysine [Aloc-Lys]) was introduced to LAMP2A at the position specified by the *amber* codon UAG, during translation with an *amber* suppressor tRNA and engineered aminoacyl-tRNA synthetases that can charge tRNA with *p*Bpa or Aloc-Lys, respectively. Upon UV irradiation, the carbonyl carbon within the side chain of *p*Bpa (orange in Fig. 2A) forms a covalent bond with an atom in its vicinity, at least for some duration, leading to an intermolecular linkage between the associated molecules. This crosslinking between two molecules verifies that their interaction is direct, and not mediated by other molecules. The side chain of Aloc-Lys (green in Fig. 2A) is bulkier than those of other natural amino acids. If the introduction of Aloc-Lys to a position on the surface of the molecule reduces the coimmunoprecipitation efficiency by disrupting the homophilic interaction, then this position may be located within the interface.

**Figure 2.**
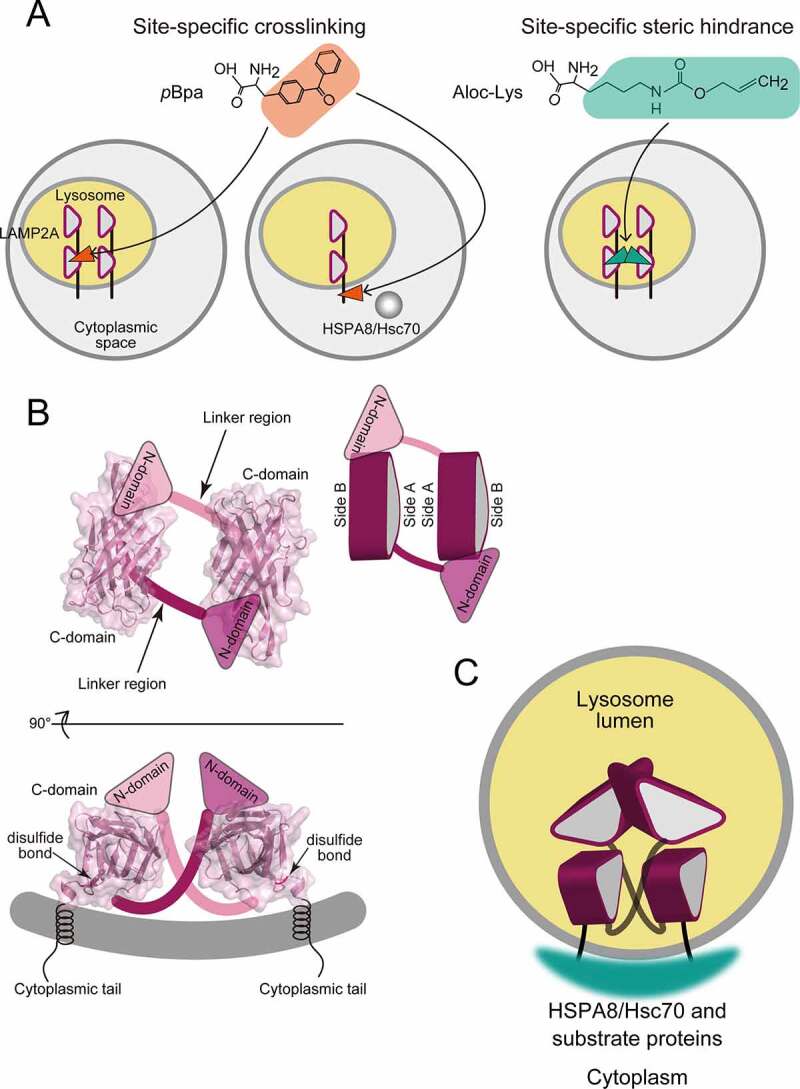
Schematic presentations of the homophilic interaction of LAMP2A and the interaction between the cytoplasmic tails of LAMP2A molecules with the HSPA8-substrate protein complex on the lumenal membrane. (**A**) The two types of experiments in our study. (**B**) The C-domains of two LAMP2A proteins in the side-A-faced orientation in two perpendicular views. Putative locations of the N-domains are indicated by triangles. (**C**) Proposed multi-point contacts between LAMP2A molecules and the chaperone-substrate complex.

The site-specific crosslinking demonstrated that the homophilic interaction of LAMP2A is direct, and the C-domain contributes to it. Combined with the steric hindrance experiments, the LAMP2A molecules on the lumenal membrane apparently orient each side A of the β-prism in the C-domain toward the other, in the cases of mouse and human LAMP2A (Fig. 2B) [1]. Because the N terminus of the C-domain, where the linker region is attached, is located near the lumenal membrane in our model, we consider that the linker region is perpendicular to the membrane along the C-domain, and thus restricts the interaction between the N-domains to the side-A-faced arrangement of the C-domains (Fig. 2B).

We also introduced *p*Bpa within the cytoplasmic tail of LAMP2A and detected its crosslinking with HSPA8, revealing this direct interaction. Subsequently, we found that the N-domain truncation of LAMP2A reduces the amount of coimmunoprecipitated HSPA8 [1].

Why was a smaller amount of HSPA8 retrieved by LAMP2A with the N-domain truncation, although the cytoplasmic tail of LAMP2A remains intact? We consider that a few HSPA8 molecules, associated with their substrate proteins, simultaneously interact with several cytoplasmic tails of LAMP2A, and this interaction requires the proper presentation of the cytoplasmic tails on the lysosome membranes (Fig. 2C).

The cytoplasmic tail of LAMP2A is preceded by the unique α-helix of the C-domain, fixed to the β-prism by a disulfide bond (Fig. 2B). Thus, the assembly of the LAMP2A molecules in the lumen determines the positions of the cytoplasmic tails. Accordingly, the truncation of the N-domain of LAMP2A molecules likely affects the interaction with HSPA8 by changing the homophilic LAMP2A interactions.

Our study suggested the close relationship between the homophilic interactions of LAMP2A within the lysosomal lumen and the recruitment of substrate proteins by HSPA8 to the cytoplasmic surface of the lysosome in CMA. We propose that the LAMP2A assembly in the lysosomal lumen may be a therapeutic target to manipulate the CMA activity.
